# Dissemination of OXA-23 carbapenemase-producing Proteus mirabilis and Escherichia coli is driven by transposon-carrying lineages in the UK

**DOI:** 10.1099/mgen.0.001502

**Published:** 2025-09-11

**Authors:** Roxana Zamudio, Karen Osman, Rachel Pike, Aiysha Chaudhry, Danièle Meunier, Nicole Stoesser, Rebecca Stretch, Jane F Turton, David Williams, Katie L. Hopkins

**Affiliations:** 1 AMR and HCAI Division, UK Health Security Agency (UKHSA), London, UK; 2 Antimicrobial Resistance and Healthcare Associated Infections (AMRHAI) Reference Unit, Public Health Microbiology – Reference Microbiology Division, UK Health Security Agency (UKHSA), London, UK; 3 Nuffield Department of Medicine, University of Oxford, Oxford, UK; 4 Oxford University Hospitals NHS Foundation Trust, Oxford, UK; 5 NIHR Oxford Biomedical Research Centre, Oxford University Hospitals NHS Foundation Trust, John Radcliffe Hospital, Oxford, UK; 6 NIHR Health Protection Research Unit in Healthcare Associated Infections and Antimicrobial Resistance at University of Oxford, Oxford, UK

**Keywords:** carbapenemase, *Escherichia coli*, OXA-23, *Proteus mirabilis*, transposon

## Abstract

Carbapenem-resistant *Enterobacterales* are a significant threat to global public health. Here, we characterize *bla*_OXA-23_-positive *Proteus mirabilis* (*n*=8) and *Escherichia coli* (*n*=3) isolates from human clinical samples collected between 2021 and 2024 in the UK. Whole-genome sequencing (WGS) was used to generate data, and a phylogenetic tree inferred from SNPs filtered for recombination was constructed to assess the genomic relatedness among the isolates. To provide an international context, we included publicly available genomes. Short-read mapping to a reference genome enabled reconstruction of the genomic neighbourhood around *bla*_OXA-23_. Minimum inhibitory concentration (MIC) determination was performed using broth microdilution and results interpreted using the European Committee on Antimicrobial Susceptibility Testing (EUCAST) guidelines. UK *P. mirabilis* isolates belonged to ST142 and formed a sub-clade descending from an ancestral cluster of French isolates with relatively few SNPs between them (9–39). *E. coli* ST38 isolates harboured *bla*_OXA-23_ and showed close genetic relatedness (12–15 SNPs) among themselves. In *P. mirabilis*, *bla*_OXA-23_ was associated with transposon Tn*6703*, while *E. coli* harboured a novel composite transposon, designated Tn*7816*, bordered by two copies of IS*26* and with three copies of *bla*_OXA-23_. *bla*_OXA-23_ was integrated into the chromosome in all isolates. All isolates were resistant to amoxicillin/clavulanic acid (>32 mg l^−1^) and with meropenem MICs above the EUCAST screening cut-off (0.5–1 mg l^−1^). In conclusion, UK *bla*_OXA-23_-positive *P. mirabilis* isolates belong to the same clonal lineage (ST142) previously reported in Belgium, Germany, Switzerland and France, suggesting introduction of this lineage into the UK. This is the first report of an *E. coli* ST38 lineage with chromosomally encoded *bla*_OXA-23_ located within a novel transposon Tn*7816*. WGS plays an important role in identifying the mechanism(s) of transmission of emerging carbapenemase genes.

**Impact Statement**

Most diagnostic assays primarily focus on the detection of the ‘big 5’ carbapenemase gene families (KPC, OXA-48-like, NDM, VIM and IMP), which are globally dominant in *Enterobacterales*. OXA-23-like carbapenemase genes are predominantly identified in *Acinetobacter baumannii* and their presence in *Enterobacterales* is likely underestimated. In this study, we report the identification of *bla*_OXA-23_ in *Proteus mirabilis* and *Escherichia coli* isolated from UK clinical samples following incorporation of *bla*_OXA-23-like_ as a target in the multiplex PCR assay used to screen all Gram-negative bacteria referred to the UK’s national reference laboratory for investigation of carbapenem resistance. To enhance our understanding of the genomic epidemiology of *bla*_OXA-23_, we utilized short-read sequencing to characterize all isolates and long-read-only assembly, polished with short reads to determine the genomic context of *bla*_OXA-23_ in *E. coli*. Whole-genome sequencing (WGS) analysis identified that *bla*_OXA-23_-positive *P. mirabilis* isolates were closely related to those previously reported in Europe and uncovered a novel transposon associated with *bla*_OXA-23_ in *E. coli*. In addition, *bla*_OXA-23_ was chromosomally located in all isolates, with the potential for stable vertical inheritance. The results of this study highlight the need to further characterize *Enterobacterales* isolates suspected of carbapenemase production but negative for the ‘big 5’ carbapenemase gene families and further demonstrate the role of WGS in characterizing bacterial strains and mobile genetic elements associated with the emergence and transmission of antimicrobial resistance mechanisms.

## Data Summary

Illumina short-read, contigs and minimum inhibitory concentration (if available) data for *Proteus mirabilis* (*n*=8) and *Escherichia coli* (*n*=3) *bla*_OXA-23_-positive isolates from the UK are available in the European Nucleotide Archive database under BioProject PRJEB80458. The Illumina polished version of the long-read-only (Nanopore) assembly for *E. coli* 1697008 (ES1) isolate is accessible under the GenBank accession number OZ204848. Nanopore reads for *E. coli* 1697008 (ES1) isolate are accessible under the BioSample SAMN37200062, SRA SRR34389851 and BioProject PRJNA1010831. The accession numbers for each genome and the metadata generated in this study are provided in Table 1.

**Table 1.** Accession numbers, metadata and antimicrobial susceptibility testing data for *P. mirabilis* (*n*=8) and *E. coli* (*n*=3) isolates obtained from human clinical samples in the UK The table displays accession numbers for *bla*_OXA-23_-positive isolates under the BioProject PRJEB80458 and the MIC values (in mg l^−1^) for 12 antibiotics.

Isolate ID	Biosample	Species	Isolation source	Year	ST	AMP	AUG	PTZ	AMK	GEN	CIP	CTX	CAZ	CPM	ERP	MEM	IM	All AMR determinants
1165744	SAMEA116133959	*P. mirabilis*	Urine	2021	ST142	>32	>32	16	4	>16	≤0.125	≤0.25	≤0.25	≤0.5	0.25	1	4	*bla*_OXA-23_ *, aac(3)-IIe, aadA1, aph(3'')-Ib, aph(3')-Ia, aph(6)-Id, catA, dfrA1, sat2, sul2, tet(J)*
1165743	SAMEA116133960	*P. mirabilis*	Urine	2022	ST142	>32	>32	16	4	>16	≤0.125	≤0.25	≤0.25	≤0.5	0.25	1	8	*bla*_OXA-23_ *, aac(3)-IIe, aadA1, aph(3'')-Ib, aph(3')-Ia, aph(6)-Id, catA, dfrA1, floR, sat2, sul2, tet(J)*
1875382	SAMEA116133962	*P. mirabilis*	Wound and pus from the knee	2022	ST142	>32	>32	16	≤1	>16	≤0.125	≤0.25	≤0.25	≤0.5	nt	0.5	8	
1158158	SAMEA116133963	*P. mirabilis*	Urine	2023	ST142	nt	nt	nt	nt	nt	nt	nt	nt	nt	nt	nt	nt	
1217625	SAMEA116133964	*P. mirabilis*	Urine	2023	ST142	>32	>32	8	2	>16	≤0.125	≤0.25	≤0.25	≤0.5	1	0.5	8	
1316870	SAMEA116133965	*P. mirabilis*	Rectal swab	2023	ST142	nt	nt	nt	nt	nt	nt	nt	nt	nt	nt	nt	nt	
1386372	SAMEA116133966	*P. mirabilis*	Urine	2023	ST142	>32	>32	4	2	>16	≤0.125	≤0.25	≤0.25	≤0.5	≤0.125	0.5	4	
1495047	SAMEA116133968	*P. mirabilis*	Urine	2024	ST142	nt	nt	nt	nt	nt	nt	nt	nt	nt	nt	nt	nt	
1697008*	SAMEA116133961	*E. coli*	Rectal swab	2022	ST38	>32	>32	>64	≤1	≤0.25	≤0.125	1	≤0.25	1	1	1	1	*bla*_OXA-23_*, acrF, blaEC, cyaA*_S352T, *emrD*, *glpT*_E448K
1473365	SAMEA116133967	*E. coli*	Rectal swab	2024	ST38	>32	>32	>64	1	0.5	≤0.125	1	≤0.25	2	2	0.5	1	
1539851	SAMEA116133969	*E. coli*	Rectal swab	2024	ST38	>32	>32	>64	8	4	≤0.125	1	≤0.25	2	1	0.5	1	

*The Illumina polished version of the long-read-only complete chromosome for the 1697008 (also known as ES1) isolate is available under the assembly GCA_964341145 and GenBank OZ204848.

AMK, amikacin; AMP, ampicillin; AUG, amoxicillin/clavulanic acid; CAZ, ceftazidime; CIP, ciprofloxacin; CPM, cefepime; CTX, cefotaxime; ERP, ertapenem; GEN, gentamicin; IM, imipenem; MEM, meropenem; PTZ, piperacillin/tazobactam.;

## Introduction

Carbapenem-resistant *Enterobacterales* were designated as critical priority pathogens by the World Health Organization because of limited treatment options and widespread prevalence [1]. Carbapenem resistance in *Enterobacterales* is primarily driven by the acquisition of one or more of the ‘big 5’ carbapenemase genes; the *β*-lactamases of Ambler class A (*bla*_KPC_), class B (*bla*_NDM_, *bla*_VIM_, *bla*_IMP_) and class D (*bla*_OXA-48-like_) [2]. In contrast, *bla*_OXA-23_ is commonly present in *Acinetobacter baumannii* and has been disseminated globally [3] but has only been occasionally reported in *Enterobacterales*. Chromosomally encoded *bla*_OXA-23_ in *Proteus mirabilis* was first detected in 1996 in France, and since then, isolates have been reported in various European countries in clinical [4,10] and animal samples [5]. In contrast, reports of *bla*_OXA-23_ in *Escherichia coli* are very rare, with the first clinical case identified in Singapore [11], and 14 subsequent cases documented in India between 2013 and 2014 [12]. Since 2020, the UK’s national reference laboratory has screened all Gram-negatives submitted for investigation of carbapenem resistance with a multiplex PCR including *bla*_OXA-23-like_, *bla*_OXA-40-like_, *bla*_OXA-51-like_ and *bla*_OXA-58-like_ families. Here, we describe the genomic characterization of the first UK *P. mirabilis* and *E. coli* isolates harbouring *bla*_OXA-23_.

## Methods

### Bacterial isolates and antimicrobial susceptibility testing

Isolates were referred to the UK Health Security Agency Antimicrobial Resistance and Healthcare Associated Infections (AMRHAI) Reference Unit for investigation of carbapenem resistance following recovery from clinical human specimens (urine, rectal swab and wound) collected in the UK between 2021 and 2024. Antimicrobial susceptibility testing was performed using broth microdilution against AMRHAI’s standard antibiotic panel and results interpreted according to the European Committee on Antimicrobial Susceptibility Testing (EUCAST) clinical breakpoints version 14.0 [13].

### Whole-genome sequence and genome collection

Paired-end short-read sequencing was performed using Illumina technology. The methods for DNA extraction and whole-genome sequencing (WGS) were previously described [14]; briefly, DNA was extracted from RNAse-treated lysates using a QIAsymphony DSP DNA Midi Kit (QIAGEN, Hilden, Germany) and sequenced on a Hiseq 2500 instrument (Illumina, San Diego, CA, USA) using the standard 2×101 bp sequencing protocol. Additionally, for the *bla*_OXA-23_-positive 1697008 *E. coli* isolate, long-read data were available. DNA extraction for MinION sequencing was carried out using a GeneJet genomic DNA Kit (Thermo Fisher, Loughborough, UK). Note that DNA extractions for Nanopore and Illumina sequencing for the 1697008 *E. coli* isolate were performed at different times, from distinct colony picks and extracts. Long-read sequencing was performed on a minION Mk1C device (Oxford Nanopore Technologies, Oxford, UK) using an R10.4.1 flow cell following library preparation using the rapid barcoding kit SQK-RBK114.24. Basecalling of raw fast5 files was performed in real-time using Guppy v6.3.9 with the ‘high-accuracy’ model via minKNOW 22.10.7, which used an embedded graphics processing unit.

*bla*_OXA-23_-positive *P. mirabilis* genomes (*n*=56) from four previous European WGS studies [5,8] were included for international context (Table S1, available in the online Supplementary Material). These studies were selected because their WGS data were publicly available at the time of the literature search in National Center for Biotechnology Information (NCBI)/PubMed. From these studies, draft genomes were available for most isolates, while a subset of the French isolates had accessible Illumina short-read data and a complete chromosome sequence was available for one isolate from France (designated as VAC; GenBank: CP042907.1). Furthermore, 32 *bla*_OXA-23_-negative *E. coli* ST38 genomes from a previous UK study [15], with Illumina short-read availability, were included for comparative genome analysis with the *bla*_OXA-23_-positive *E. coli* ST38 (Table S1). The UK study [15] was selected because it provided a substantial number of ST38 isolates from the UK. More details on collecting WGS data from the previously cited studies and associated databases are available in the supplementary methods (see Material S1).

### Assembly, typing and screening of antimicrobial resistance determinants

Illumina reads were processed with Trimmomatic v0.39 [16] (default parameters) to eliminate adapters and poor-quality bases. Subsequently, the trimmed reads were utilized to assemble draft genomes using SPAdes v3.11.1 [17] with default parameters and the --careful option. Assemblies were evaluated using QUAST v5.2.0 [18] for summary statistics, and quality was also assessed with CheckM v1.2.2 [19], where any genomes with less than 95 % completeness and/or assembly contamination exceeding 2 % were excluded. Nanopore raw reads were trimmed for barcodes and filtered by quality score (minimum qscore 9) using minKNOW 22.10.7. SeqKit v2.9 [20] was then used to determine the quality metric of the Nanopore reads. These filtered reads were subsequently used to obtain the long-read-only assembly using Flye v2.9.1-b1780 [21] with the --nano-raw parameter. Medaka v1.7.2 was employed with default settings for one round of polishing (one iteration) using long reads. The long-read-only assembly for the 1697008 isolate (also known as ES1) yielded a complete chromosome sequence and is accessible under GenBank accession number CP133856.1. To obtain high accuracy of the 1697008 (ES1) long-read-only assembly, a further polishing step was performed using Illumina reads and Pilon v1.24 [22]. This process involved mapping the trimmed Illumina reads against the long-read-only assembly with Minimap2, followed by variant detection and correction by Pilon. A single iteration of Pilon polishing was sufficient to correct a small number of mismatches (31 SNPs and 40 indels, which represent 0.001 % of the sequence) in the long-read-only assembly. These detected variants may be attributable to the distinct DNA extract used for Illumina and Nanopore sequencing, and/or systematic variations introduced by the distinct sequencing technologies employed. The Illumina polished version of the long-read-only assembly for the 1697008 isolate is available in GenBank under accession number OZ204848. This Illumina polished version was used in subsequent analysis. Antimicrobial resistance (AMR) genes and point mutations were identified in the assemblies using AMRFinderPlus v3.12.8 [23] with database version 2024-01-31.1 [23]. Multi-locus sequence typing (MLST) schemes for *Proteus* spp. and *E. coli* were utilized to determine the sequence type (ST) of each isolate through the pMLST portal (https://pubmlst.org/). Phylotyping for *E. coli* was conducted using the ClermonTyping v20.3 [24] tool.

### Phylogenetic analysis

The genetic relatedness among the *P. mirabilis* isolates was assessed through phylogenetic analysis. Given that *de novo* assemblies were mostly available, contig sequences were converted into split-k-mer files. These split-k-mers were subsequently mapped to the VAC chromosome reference genome (GenBank CP042907.1) using SKA v1.0 (https://github.com/simonrharris/SKA) to generate a core genome multiple sequence alignment. Recombination regions were then identified and removed from this alignment using Gubbins v3.4 [25]. Then, a maximum likelihood tree was constructed based on the recombination-filtered SNP data. For this analysis, Gubbins was set to use the general time-reversible substitution model, with tree construction performed by IQ-TREE v2.4.0 [26]. Branch supports were assessed with a 1,000 ultrafast bootstrap [27] approach implemented in IQ-TREE. The recombination-corrected phylogenetic tree generated by Gubbins is scaled to substitutions per genome. The genetic relatedness for *E. coli* ST38 isolates was assessed using the same phylogenetic methods; however, a short-read mapping approach was used, given the availability of short-read data for all *E. coli* isolates. By not mixing short- and long-read sequence data in this comparative analysis, any difference in systematic error between the platforms was avoided. Snippy v4.3.6 [28] was used to map sequence reads to the *E. coli* 1697008 chromosome reference genome (Genbank OZ204848) for reconstructing the consensus sequence for each isolate. Subsequently, the consensus sequences from all isolates were processed with Gubbins, as detailed above. The *P. mirabilis* tree was rooted using the 160A10 isolate (ST185) as an outgroup, while the *E. coli* tree was midpoint rooted, as no outgroup was used. The trees were plotted alongside the metadata (country, source, year, ST and the presence/absence of AMR genes and other relevant data) using ggtree v3.12.0 [29] R [30]. The cophenetic distance was used to measure the evolutionary distance between the isolates connected by their most recent common ancestor on a phylogenetic tree. Thus, the SNP pairwise distance between isolates was obtained from the cophenetic distances. More details on the cophenetic distance analysis are available in the supplementary methods (see Material S1).

### Mapping short reads and genomic context

To assess the vehicles that potentially mobilize *bla*_OXA-23_ in *P. mirabilis* and *E. coli*, the genomic context was investigated by using Snippy v4.3.6 [28] to map sequence reads to a reference genome (as detailed above) and reconstructing the consensus sequence for each isolate (details in supplementary methods, Material S1). The consensus sequence for each isolate was annotated with Bakta v1.9.2 [31] to identify insertion sequences (ISs) and AMR genes. To ensure accurate IS annotation, ISfinder [32] (database v2025.06.06) was also used. The annotation of the transposable elements associated with *bla*_OXA-23_ in *P. mirabilis* was performed as described by Bonnin *et al.* [5]. The complete chromosome sequence available for isolate 1697008 enabled the identification of the genomic context associated with *bla*_OXA-23_ in *E. coli*. This was achieved through manual annotation based on the genetic characteristics of transposable elements [26], including the presence of ISs, terminal inverted repeat sequences and target site duplications (TSD), also known as direct repeats. TnCentral [33] was used to identify transposons similar to the ones identified in this study. Additionally, IslandViewer 4 [34] was used to identify genomic islands within the 1697008 genome. Finally, the genomic contexts of *bla*_OXA-23_ were compared using blastn v2.15.0 [35] and visualized along with their annotations using the genoPlotR v0.8.11 [36] R package.

Additionally, the copy number of the *bla*_OXA-23_-containing region was estimated by analysing the sequencing depth of coverage. The read depth at each nucleotide position was extracted from BAM files using SAMtools v1.9 [37] (with the depth -a option). To account for variable sequencing depth across samples, the individual nucleotide depths were normalized by dividing them by the mean genomic depth coverage. This normalization yielded a relative depth coverage, which directly links with the gene copy number; for instance, a normalized depth of 1.0 indicates a single copy, 2.0 suggests two copies and so on. Given that G+C content significantly influences sequencing coverage [38], the G+C content of the reference sequence was calculated using a 200-nt sliding window. Finally, to ensure the reliability of the short-read mapping approach, the proportion of properly paired mapped reads was evaluated using SAMtools v1.9 [37] (with the flagstats option).

## Results and discussion

### Clonal relatedness of OXA-23-producing *P. mirabilis* and *E. coli* isolates

**Fig. 1.**
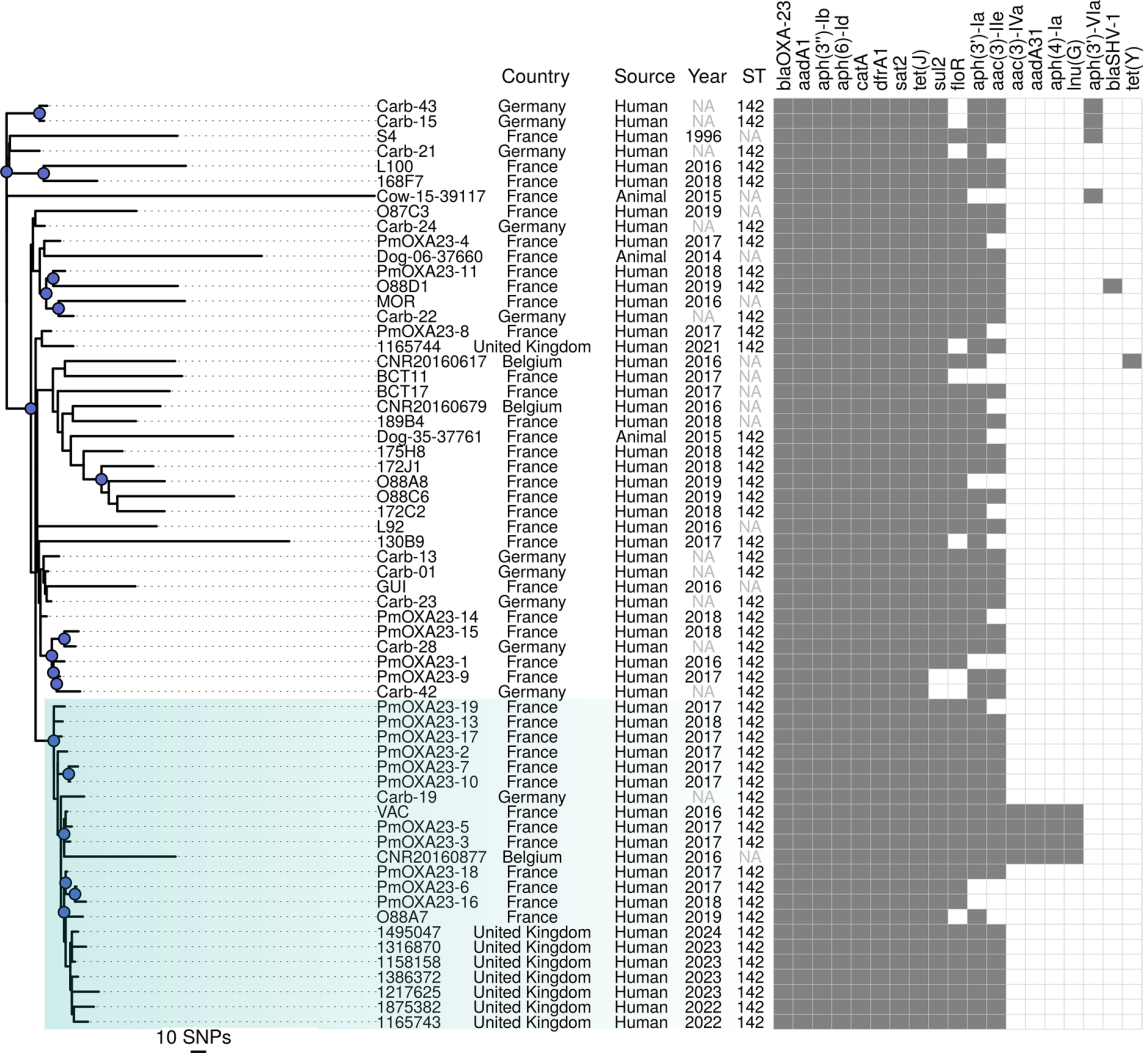
Recombination-filtered SNP-based rooted phylogeny of 62 *bla*_OXA-23_-positive *P. mirabilis* isolates. The phylogenetic relationship analysis included *bla*_OXA-23_-positive ST142 isolates from the UK (*n*=8) and isolates obtained from prior studies (*n*=54) conducted in various European countries. Next to the tree are represented the metadata such as country, source, year, ST and AMR determinants. ‘NA’ label within the year field indicated that year-related information was not available, while ‘NA’ in ST indicates that it was not feasible to establish the ST due to missing or incomplete MLST loci. This includes the absence or partial coverage (44 –86 %) of *recA* in 11 genomes, the absence of *pyrC* in one genome or partial coverage (75 –58 %) of *dnaJ* in two genomes, possibly attributable to fragmented contigs. Bootstrap support ≥95 % (blue dots) indicates well-supported clades, with the clade of interest highlighted in pale blue. Scale bars represent a phylogenetic distance of 10 SNPs.

The *bla*_OXA-23_ gene was identified in eight *P. mirabilis* and three *E. coli* (Table 1)*.* A sequencing mapping analysis of 64 OXA-23-producing *P. mirabilis* genomes against the VAC reference genome (GenBank CP042907.1) resulted in between 80.6% and 97.2 % of the reference bases being covered. From this alignment, 21,589 recombination-filtered SNPs were extracted. Sixty-two of 64 isolates belonged to ST142, while the remaining 2 isolates were ST135 and ST185. There was substantial genetic diversity observed between isolates of these different STs, with ST135 and ST185 unrelated to ST142, differing at five of the six loci. All ST142 isolates formed a main cluster with some divergence between clades (Fig. 1), with an average pairwise SNP difference of 101 between these isolates. This main ST142 lineage encompasses isolates derived from France, Belgium, Germany and the UK, as well as from diverse sources of human and animal origin.

Seven out of the eight *P. mirabilis* isolates from the UK were closely related, with a range of 6–31 pairwise SNPs, and grouped in the tree. The remaining UK isolate (1165744 in Fig. 1) was placed in a different clade, with 26 SNPs to its nearest relative, an isolate from France, but 50–65 SNPs when compared with the group of seven highly similar UK isolates. In this study, a clade was defined as a group of isolates sharing an exclusive common ancestor and supported by a bootstrap value ≥95%. The ‘clade of interest’ was specifically designated as the clade containing the seven UK isolates and 15 international isolates (of which 13 were from France), and showed a median pairwise SNP difference of 22 [interquartile range (IQR) 17 to 28] between the isolates. Within this clade of interest, the seven UK isolates formed a sub-clade descending from among the ancestral cluster of mostly French isolates from 2016 to 2019 (PmOXA23-19, PmOXA23-13, PmOXA23-17, PmOXA23-2, PmOXA23-7, PmOXA23-10, PmOXA23-5, PmOXA23-3, PmOXA23-18, PmOXA23-6, PmOXA23-16, VAC and O88A7), with pairwise SNP distances ranging from 9 to 39. A similar range of diversity has been observed and reported as closely related among French and Swiss isolates [10]. The clade of interest includes the French VAC isolate, for which a complete genome is available. The French VAC isolate differed from the seven UK isolates by 13–28 SNPs (Fig. 1). These relationships suggest that the isolates detected in the UK may have been introduced recently.

The first detection of *bla*_OXA-23_-positive *P. mirabilis* occurred in France during a study conducted between 1996 and 1999 [4]. Subsequent sporadic detections were reported in clinical samples from 2016 to 2018, also in France. Notably, the affected patients had no history of international travel, indicating possible community acquisition of *bla*_OXA-23_-positive *P. mirabilis* [7]. Similarly, no travel history was reported for any of the patients from whom UK isolates were recovered. *bla*_OXA-23_-positive *P. mirabilis* has subsequently been reported from 12 hospitals in France [6], a routine screening sample in Finland in 2014 [9], clinical samples in Germany between 2013 and 2022 [8] and in Belgium and France, including animal samples, between 2014 and 2018 [5]. More recently, *bla*_OXA-23_-positive *P. mirabilis* has been identified in clinical samples from Switzerland between 2017 and 2023 [10]. Overall, these findings indicate that the expansion of the ST142 lineage has driven the emergence of *bla*_OXA-23_-positive *P. mirabilis* in multiple countries and sources. Beyond Europe, the presence of *bla*_OXA-23_-positive *P. mirabilis* has been reported in a clinical sample from 2022 in Singapore using PCR, although WGS was not performed for this isolate [39]. Further cases of *bla*_OXA-23_-positive *P. mirabilis* from clinical samples have also been identified in Saudi Arabia (*n*=1 ST135; biosample SAMN46745596), China (*n*=1 ST135; biosample SAMN43414873) and the USA (*n*=1 ST142; biosample SAMN41612253), all collected in 2024.

Mapping reads from UK *E. coli* isolates to the *E. coli* 1697008 chromosome reference genome (GenBank OZ204848) yielded between 90.8 and 99.9% of the reference genome with at least 5× sequencing depth coverage. From this core genome alignment, 2,052 recombination-filtered SNPs were extracted. All isolates belonged to ST38 and phylogroup D. The three *bla*_OXA-23_-positive isolates from geographically distinct regions of England (East Midlands, London and South East) were closely related, with pairwise distances between 12 and 15 SNPs. In comparison, when these three *bla*_OXA-23_-positive isolates were analysed alongside available ST38 *bla*_OXA-23_-negative isolates, much larger pairwise genetic distances were observed [median (IQR) pairwise distance: 217 (209 to 224) SNPs]. To date, there have been only two previous reports of *bla*_OXA-23_ in *E. coli*: in ST4108 in Singapore (*n*=1) [11] and ST471 in India (*n*=14)^12^; however, these studies did not generate WGS data for those isolates. To the best of our knowledge, our study is the first to report the presence of *bla*_OXA-23_ in *E. coli* ST38 in Europe.

### Antimicrobial susceptibility

In addition to *bla*_OXA-23_, the *P. mirabilis* ST142 isolates were found to harbour between 8 and 15 additional antimicrobial resistance genetic determinants (AMR genes (Fig. 1, Table 1), which potentially confer resistance to eight different antimicrobial classes; nevertheless, some of these AMR genes relate to intrinsic resistance (i.e. *tet(J)* to tetracycline and *cat* to phenicol) [5].

All phenotypically tested *P. mirabilis* and *E. coli* isolates from this study were resistant to ampicillin (>32 mg l^−1^) and amoxicillin/clavulanic acid (>32 mg l^−1^). Most (6/8; 75%) isolates were also resistant to piperacillin/tazobactam (16 mg l^−1^ for *P. mirabilis* and >64 mg l^−1^ for *E. coli*). This aligns with the fact that the OXA-23 enzyme can also hydrolyse penicillin-class antibiotics [40]. All *P. mirabilis* isolates exhibited resistance to gentamicin, likely due to the presence of *aac(3)-IIe*. However, all isolates were susceptible to amikacin, ciprofloxacin, cefotaxime, ceftazidime and cefepime. This antimicrobial susceptibility testing profile aligns with findings for OXA-23-producing *P. mirabilis* from previous studies [5,10]. However, it is noteworthy that OXA-23 has demonstrated variable activity against the third-generation cephalosporins (e.g. cefotaxime and ceftazidime) [41,42]. The meropenem minimum inhibitory concentrations (MICs) of all isolates were within the susceptible range but above the EUCAST screening cut-off for investigation of suspected carbapenemase-producing *Enterobacterales* (>0.12 mg l^−1^). However, all three *E. coli* and one *P. mirabilis* isolate were resistant to ertapenem, with MIC values ranging from 1 to 2 mg l^−1^ (Table 1) [1]. The observed low level of carbapenem resistance agrees with the previous definition that OXA-23 is a weak carbapenem-hydrolysing enzyme at least in *P. mirabilis* [5,6] and that the bacterial host may impact whether OXA enzymes are capable of hydrolysing carbapenems [43].

### Composite transposon harbouring *bla*_OXA-23_ in *P. mirabilis* ST142

**Fig. 2.**
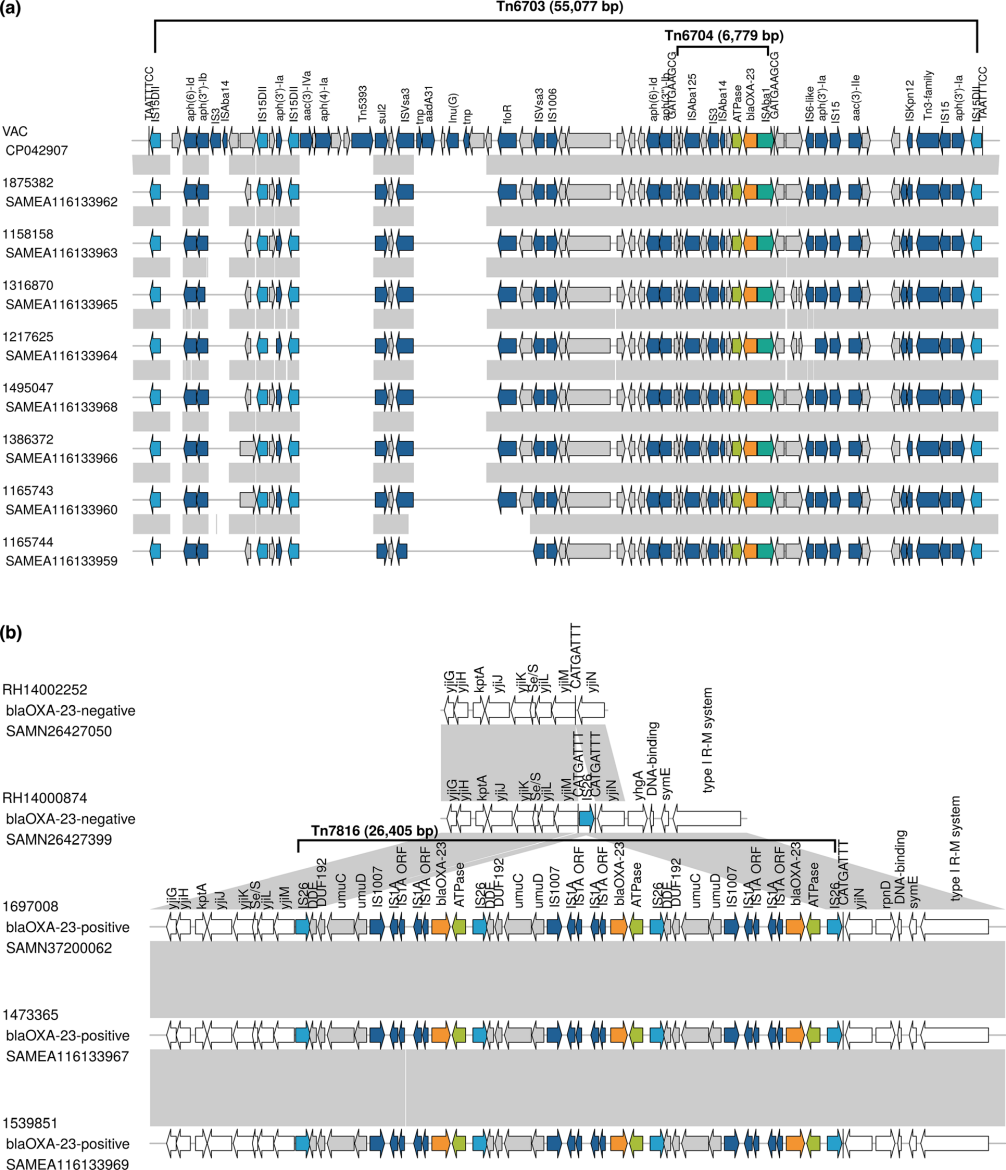
Genomic context associated with *bla*_OXA-23_ in the UK isolates. (**a**) Composite transposon Tn*6703* is present in *P. mirabilis* ST142 isolates from the UK (*n*=8) and France (with the VAC isolate as a reference), carrying AMR genes, and specifically Tn*6704*, which harbours *bla*_OXA-23_. Boundaries of Tn*6704* are indicated by the 9-bp TSD GATGAAGCG and for Tn*6703* by the 8-bp TSD TAATTTCC. (**b**) A novel composite transposon Tn*7816* associated with *bla*_OXA-23_ in *bla*_OXA-23_-positive *E. coli* ST38 in UK isolates (*n*=3). The two *bla*_OXA-23_-negative isolates yielded contig-level sequence assemblies, where the operon *yjiGHJKLMN* and genetic configuration *yjiGHJKLM*–IS*15DIV–yjiN* were in a single contig, respectively. Genes are represented by arrows indicating the direction of transcription. *bla*_OXA-23_ is indicated by the orange arrow, ATPase by the light green arrow, IS*Aba1* by the dark green arrow, IS*15DII* and IS*26* by the light blue arrows, other insertion sequences and additional AMR genes are indicated by the dark blue arrows, other genes within the transposon by the grey arrows and other genes outside the transposon by the white arrows. Grey areas between the linear plots represent nucleotide sequence identity (mostly >90 %).

The neighbouring genes of *bla*_OXA-23_ were annotated in the flanking sequences, obtained using a short-read mapping approach with the French VAC *P. mirabilis* genome as a reference (Fig. S1). As previously reported in the French VAC genome [5], *bla*_OXA-23_ is located within the transposon Tn*6704* (6,779 bp of length) with a gene configuration IS*Aba125*–IS*3*–IS*Aba14*–ATPase–*bla*_OXA-23_–IS*Aba1*–IS*4*, which is part of a larger composite transposon Tn*6703* (55,077 bp of length) [5] (Fig. 2A). The association between IS*Aba1* and *bla*_OXA-23_ has been well-documented in both *A. baumannii* [3] and *P. mirabilis* [5,7, 9, 10], and IS*Aba1* is known to provide a promoter sequence that facilitates the expression of *bla*_OXA-23_ [44].

When comparing the VAC reference genome with the UK genomes, Tn*6703* was found to harbour six additional AMR genes in addition to *bla*_OXA-23_ in all genomes, conferring resistance to aminoglycosides [two copies each of *aph(6)-Id* (also known as *strB*) and *aph(3″)-Ib* (also known as *strA*); three copies of *aph(3′)-Ia* and one *aac(3)-IIe*], sulfonamides (*sul2*) and phenicol (*floR*, except one UK isolate). The VAC reference genome carried an additional four AMR genes [aminoglycosides: *aac(3)-IVa*, *aph(4)-Ia* and *aadA31*; lincosamides: (*lnu*(G)] that were absent from all UK isolates (Fig. S1). These findings align with previously observed variability in the gene content of the composite transposon Tn*6703* among the French [5] and Swiss [10] isolates. This latter study grouped the Swiss isolates into ‘group 1’ and ‘group 2’ based on AMR content [10]. Seven of our UK isolates show AMR content consistent with ‘group 1’, while the remaining UK isolate aligned with ‘Group 2’. Minor discrepancies in AMR annotation were observed, likely due to the use of different AMR detection databases.

### Novel composite transposon associated with *bla*_OXA-23_ in *E. coli* ST38

The genomic context of *bla*_OXA-23_ in *E. coli* ST38 from the UK was assessed using the 1697008 complete *E. coli* chromosome as a reference in the short-read mapping analysis. To further elucidate the transposon integration within the chromosome of the ST38 lineage, *bla*_OXA-23-_negative *E. coli* ST38 genomes were also included in this study for comparative analysis (Fig. S2). This analysis revealed the integration of IS*26* (which included left and right inverted terminal repeats sequence 14 bp GGCACTGTTGCAAA) within the chromosome of *bla*_OXA-23-_negative ST38 RH14000874 isolate, flanked by the 8-bp TSD sequence CATGATTT. This integration disrupted the *yjiGHJKLMN* operon, resulting in the formation of a new genetic configuration, *yjiGHJKLM*–IS*26–yjiN*, in these isolates (Fig. 2b and Fig. S2). In the 1697008 isolate, along with the other two *bla*_OXA-23_-positive *E. coli*, *bla*_OXA-23_ was identified within a genomic island that corresponded to the boundaries of a novel transposon, which was named Tn*7816* according to the Transposon Registry database (https://transposon.lstmed.ac.uk/tn-registry). Tn*7816* is a composite transposon bordered by IS*26* (which belongs to the IS*6* family transposase [45] at both ends, flanked by an 8-bp TSD sequence CATGATTT only at the right end (Fig. 2). This structure suggests a deletion of the left-end TSD sequence during transposition and rearrangement events. This composite transposon was integrated into the *yjiGHJKLMN* operon, causing a disruption similar to that observed in the RH14000874 isolate, although in that case, the operon disruption was caused by an IS*26* element. The presence of IS*26* is noteworthy as it has been previously linked to AMR genes [46]. Tn*7816* carried three copies of *bla*_OXA-23_, each embedded within the specific genetic context: IS*26*–DDE–DUF192–*umuC–umuD*–IS*1007*–IS*1A*–IS*1A* ORF–IS*1A*–IS*1A* ORF–*bla*_OXA-23_–ATPase (Fig. 2). While this configuration differs from those previously reported in *A. baumannii* [3] and *P. mirabilis* [5], the presence of IS*1A* linked with *bla*_OXA-23_ is consistent with a previous report of plasmid-borne *bla*_OXA-23_ in *E. coli* [11]. However, previous studies on *E. coli* ST4108 and ST471 were unable to characterize the detailed genetic context of plasmid-borne *bla*_OXA-23_ due to PCR assay limitations [11,12]. With all of this, we can hypothesize that *E. coli* ST38 might have acquired an OXA-23-encoding plasmid followed by integration of *bla*_OXA-23_ into the chromosome via mobile genetic elements, as reported here. A blastn search (https://blast.ncbi.nlm.nih.gov/Blast.cgi) against the NCBI core nucleotide database (core_nt) identified *E. coli* ST457 isolate CE1628 (GenBank CP053852.1) from Australia (avian origin) with partial alignment with the transposon Tn*7816* sequence. However, in that genome, the genomic context of *bla*_OXA-23_ [IS*26* (inverse orientation)–DUF192–*umuC–umuD*–IS*1007*–IS*1*–IS*1A–bla*_OXA-23_–ATPase] and chromosomal location differed from the one investigated here. Specifically, the CE1628 isolate contains only a single copy of each *bla*_OXA-23_, IS*1* and IS*1A*. Furthermore, TnCentral results showed that only the *bla*_OXA-23_ and ATPase, and no other genes associated with Tn*2006*, aligned with the sequence of the composite transposon Tn*7816*. Consequently, this study presents the first comprehensive characterization of this unique *bla*_OXA-23_ genetic context within *E. coli*.

IS*26* has been previously associated with the rearrangement of an IncF plasmid carrying *bla*_NDM-5_ [47]. Furthermore, another study [48] highlighted the critical role of IS*26* in gene duplication and amplification, specifically multiple copies of IS*26* and IS*1* have been associated with the amplification of the *bla*_CMY-2_-containing region and consequently increased resistance to cefotaxime in *Salmonella enterica* serovar Typhimurium. In our study, the observation of three copies of *bla*_OXA-23_ in the *E. coli* 1697008 chromosome could potentially be attributed to the presence of four copies of IS*26* and three copies of IS*1A*. We analysed the normalized depth coverage of the *bla*_OXA-23_-containing regions in our *E. coli* isolates to identify patterns of amplification. Our findings showed that the mean copy number for the background region (surrounding genes outside of the transposon) was 1, and for Tn*7816,* it ranged from 1.0 to 1.7. Consequently, these data suggest no significant alterations in mean depth coverage across Tn*7816* compared with the background (Fig. S3). This result is in concordance with a previous study [48] that defined amplified regions as those with a mean relative depth coverage exceeding five times the background coverage, a condition not observed in our *E. coli* isolates. We also examined the normalized depth coverage across Tn*6703*, which harbours *bla*_OXA-23_ in *P. mirabilis*. Certain regions within Tn*6703* exhibited lower depth coverage than the background (Fig. S4). This discrepancy may be attributed to the high GC content (>65 %) of some genes within Tn*6703*. G+C content >65 % has been previously correlated with reduced sequencing depth [38]. Finally, short-read mapping to a reference genome enabled reconstruction of the transposon carrying the *bla*_OXA-23_ in *P. mirabilis* and *E. coli*. The accuracy of this mapping is supported by the high proportion of properly paired reads (98.29 –99.96 %), indicating that both paired-end reads were mapped correctly to the reference genome with the expected orientation and insert size.

In conclusion, our study underscores the significant role of transposon-carrying lineages in the emergence, dissemination and persistence of chromosomally encoded *bla*_OXA-23_ in *P. mirabilis* and *E. coli*. Our findings provide key insights into three major aspects. First, the wide distribution of *bla*_OXA-23_ in *P. mirabilis* across multiple sources and countries, including the UK, France and Germany, suggests international transmission of the *bla*_OXA-23_-positive *P. mirabilis* ST142 lineage. No epidemiological link could be established between the French, German and UK isolates, as they were collected in different years, and none of the UK patients had a history of recent travel abroad. However, the close genetic relatedness between *P. mirabilis* ST142 isolates from the UK and France, along with the earlier observations within France, suggest a possible introduction of this lineage into the UK. Second, the association between the previously characterized transposon Tn*6704*, which includes IS*Aba1*, and the composite transposon Tn*6703*, which contains IS*15DII*, with *bla*_OXA-23_ in *P. mirabilis*, has been established. However, a notable variability in the gene content within Tn*6703* was observed, which may be attributed to the dynamic nature of transposable elements. Third, the emergence of *bla*_OXA-23_ in an *E. coli* ST38 lineage in the UK was facilitated by a novel composite transposon Tn*7816* characterized by the presence of IS*1A*, IS*26* and three copies of *bla*_OXA-23_. Together, these observations highlight the role of WGS in characterizing novel carbapenemase producers and associated mobile genetic elements, which may inform the development of interventions to mitigate the spread of antimicrobial resistance.

Further studies are needed to monitor if *bla*_OXA-23_ becomes more widely established among *Enterobacterales*; however, surveillance is likely to be hampered by the lack of coverage of *bla*_OXA-23_ in commercial molecular assays [8], which instead focus on the ‘big 5’ carbapenemase gene families. As per the UK Standards for Microbiological Investigations [49], suspect *Enterobacterales* isolates exhibiting resistance to amoxicillin/clavulanate and/or reduced susceptibility to meropenem (MIC >0.12 mg l^−1^) but negative for the ‘big 5’ carbapenemase gene families should be referred to the AMRHAI Reference Unit for further investigation.

## Supplementary material

10.1099/mgen.0.001502 Uncited Supplementary Material 1.

## Abbreviations

AMR: antimicrobial resistance
EUCAST: European Committee on Antimicrobial Susceptibility Testing
IQR: interquartile range
ISs: insertion sequences
MICs: minimum inhibitory concentrations
MLST: multi-locus sequence typing
ST: sequence type
TSD: target site duplication
WGS: whole-genome sequencing
