# Supplementary material for: Dissemination of OXA-23 carbapenemase-producing Proteus mirabilis and Escherichia coli is driven by transposon-carrying lineages in the UK
Source: Microb Genom. 2025 Sep 11;11(9):001502. doi: 10.1099/mgen.0.001502 (PMC12426204; doi:10.1099/mgen.0.001502)
Supplement: Uncited Supplementary Material 1. [file mgen-11-01502-s001.pdf]

Supplementary material

Dissemination of OXA-23 carbapenemase-producing *Proteus mirabilis* and *Escherichia coli* is driven by transposon-carrying lineages in the UK

Roxana Zamudio[1], Karen Osman[1], Rachel Pike[2], Aiysha Chaudhry[2], Danièle Meunier[1,2], Nicole Stoesser[3,4,5,6], Rebecca Stretch[1], Jane F Turton[1,2], David Williams[1], Katie L Hopkins[1,2*]

[1]AMR and HCAI Division, UK Health Security Agency (UKHSA), London, United Kingdom
[2]Antimicrobial Resistance and Healthcare Associated Infections (AMRHAI) Reference Unit, Public Health Microbiology – Reference Microbiology Division, UK Health Security Agency (UKHSA), London, United Kingdom
[3]Nuffield Department of Medicine, University of Oxford, Oxford, United Kingdom
[4]Oxford University Hospitals NHS Foundation Trust, Oxford, United Kingdom
[5]NIHR Oxford Biomedical Research Centre, Oxford University Hospitals NHS Foundation Trust, John Radcliffe Hospital, Oxford, United Kingdom
[6]NIHR Health Protection Research Unit in Healthcare Associated Infections and Antimicrobial Resistance at University of Oxford, Oxford, United Kingdom

*Corresponding author: katie.hopkins@ukhsa.gov.uk

TABLE OF CONTENTS

Whole genome sequence data 2
SNP pairwise distance 2
Consensus sequence obtained by short-read mapping 2
SUPPLEMENTARY FIGURES 4
Supplementary Figure S1. Mapping short-reads for ST142 isolates against the reference VAC *bla*$_{OXA-23}$-positive *P. mirabilis* genome 4
Supplementary Figure S2. Mapping short-reads for ST38 isolates against the reference 1697008 (also known as ES1) *bla*$_{OXA-23}$-positive *E. coli* genome 6
Supplementary Figure S3. Normalized depth coverage of short-reads and GC content of *bla*$_{OXA-23}$ genomic context in three UK *E. coli* isolates 8
Supplementary Figure S4. Normalized depth coverage of short-reads and GC content of *bla*$_{OXA-23}$ genomic context in eight UK *P. mirabilis* isolates. 9
SUPPLEMENTARY TABLE 10
Supplementary Table S1. List of *P. mirabilis* (n=56) and *E. coli* (n=32) genomes from previous studies, including accession numbers and metadata. 10
SUPPLEMENTARY REFERENCES 13

SUPPLEMENTARY METHODS

Whole genome sequence data

This study generated Illumina short-reads for *bla*$_{\text{OXA-23}}$-positive *P. mirabilis* (n=8) and *E. coli* (n=3) isolates from the UK. In regards the publicly available genomes, the fastq files for French *bla*$_{\text{OXA-23}}$-posititve *P. mirabilis*[1] (Bioproject PRJNA490489), and UK *bla*$_{\text{OXA-23}}$-negative *E. coli* ST38[2] (Bioproject PRJNA812750) isolates were accessible and downloaded from the European Nucleotide Archive (ENA). While contigs files were available for *bla*$_{\text{OXA-23}}$-positive *P. mirabilis* from France and Belgium[3] (Bioproject PRJNA521327), France[4] (Bioproject PRJNA780406) and Germany[5] (Bioproject PRJNA915754). These contigs files were downloaded from NCBI/GenBank. A complete chromosome sequence was publicly available for a *bla*$_{\text{OXA-23}}$-positive *P. mirabilis* VAC isolate (Genbank CP042907.1) from France[3.]

For isolate 1697008 (ES1), Nanopore raw reads had 58,997 total reads, 422,960,791 total bases, a mean read length of 7,169 bp, an N50 of 9,540 bp, and a mean quality score of 13.4. After filtering, the quality of the filtered reads was: 57,359 total reads, 409,274,027 total bases, a mean read length of 7,135 bp, an N50 of 9,491 bp, and a mean quality score of 13.6.

SNP pairwise distance

The genetic distance among *bla*$_{\text{OXA-23}}$-positive *P. mirabilis* isolates was assessed by calculating the cophenetic distance by using the `cophenetic()` function available in the stats package from R[6]. The cophenetic distance measures the evolutionary distance between pair of taxa (or 'tips') on a phylogenetic tree. It is calculated by measuring the length of the branch connecting their most recent common ancestor (MRCA)[7]. Similarly, the genetic distance among the *E. coli* isolates were assessed as described above.

Consensus sequence obtained by short-read mapping

The short-reads were mapped to a reference genome to obtain the consensus sequence for each isolate. A complete chromosome of the VAC isolate (Genbank CP042907.1) from France[3] served as a reference for *bla*$_{\text{OXA-23}}$-positive *P. mirabilis* isolates (UK n=8 and France n=18), and the complete chromosome of 1697008 (ES1) isolate (GenBank OZ204848) from the UK was used as a reference for *E. coli* ST38 isolates (*bla*$_{\text{OXA-23}}$-positive n=3 and *bla*$_{\text{OXA-23}}$-negative n=32; all isolates from the UK). Short-read data were mapped against the reference using Snippy v4.3.6[8] including the settings `--mincov 5 --mapqual 0`, which allowed for a minimum depth coverage of 5x

and retrieval of sequences from multi-mapping regions. This methodology enabled the acquisition of a consensus sequence for each isolate individually. These individual sequences were aligned using Snippy-core to achieve a core genome alignment. The genomic neighbourhood around *bla*$_{OXA-23}$ gene were identified and visually presented as a heatmap adjacent to the phylogenetic tree. The consensus sequences were annotated with Bakta v1.9.2[9] and used for the analysis of the genomic context of *bla*$_{OXA-23}$ described in the main text.

SUPPLEMENTARY FIGURES

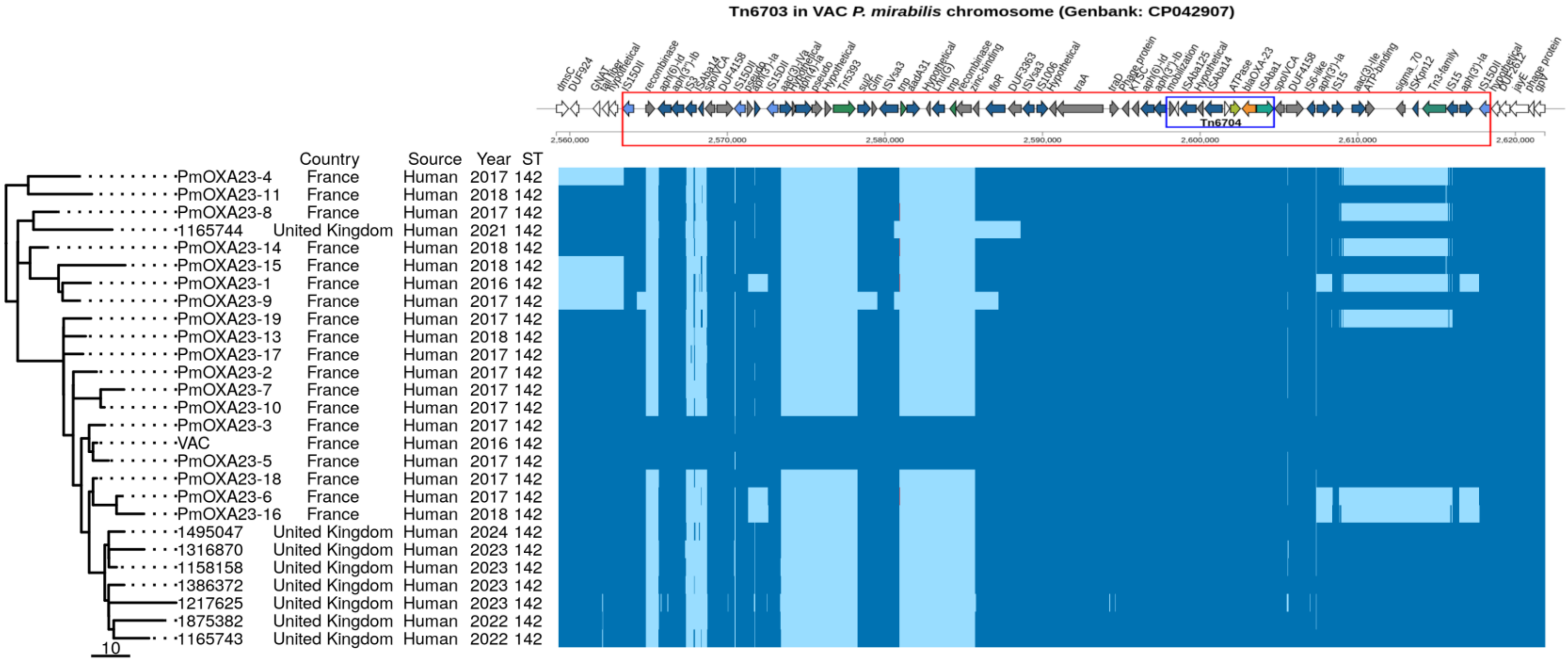

Supplementary Figure S1. Mapping short-reads for ST142 isolates against the reference VAC *bla*$_{OXA-23}$-positive *P. mirabilis* genome. Recombination-filtered SNP-based phylogeny of 27 ST142 *bla*$_{OXA-23}$-positive *P. mirabilis* genomes. The UK isolates were obtained from our study, while the French isolates were from Potron et al. 2019[1]. The accompanying metadata alongside the phylogenetic tree contains information on country, source, year, and sequence type (ST). The heatmap represents the genomic neighbourhood around the *bla*$_{OXA-23}$ obtained through the short-read mapping approach, which includes the composite transposon Tn*6703* and five genes upstream and downstream. In the heatmap, dark blue represents an identical sequence to the reference, while light blue indicates an absence of the sequence. Annotations at the top of the heatmap are represented by coloured arrows, with *bla*$_{OXA-23}$ represented in orange, ATPase in light green, IS*Aba1* in dark green, IS*15DII* in light blue, other insertion sequences and additional

antimicrobial resistance (AMR) genes in dark blue, other genes in grey, and the five genes located upstream and downstream of Tn*6703* in white. The transposons Tn*6703* and Tn*6704* are visually indicated by red and blue rectangles, respectively.

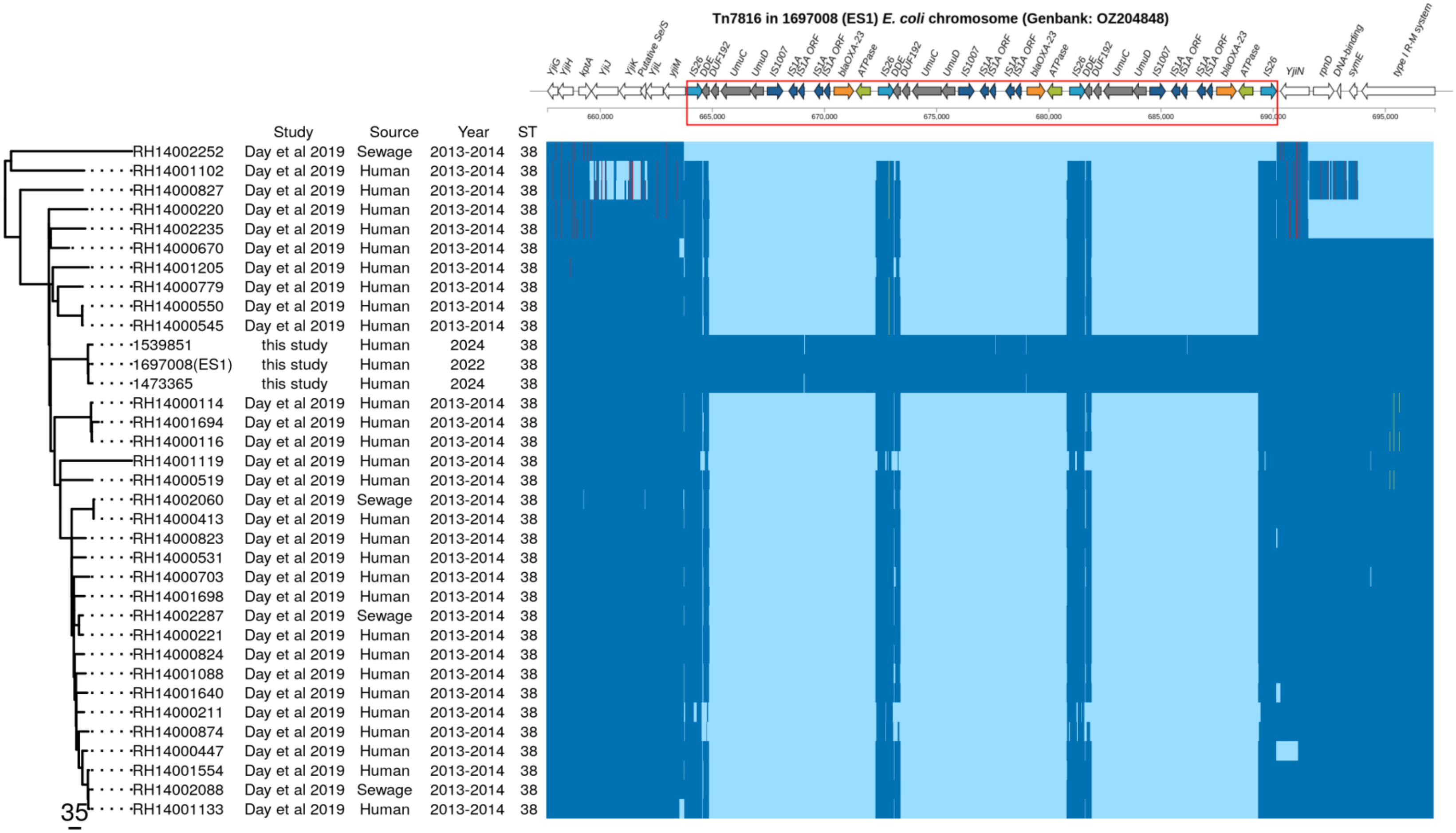

Supplementary Figure S2. Mapping short-reads for ST38 isolates against the reference 1697008 (also known as ES1) *bla*$_{OXA-23}$-positive *E. coli* genome. Recombination-filtered SNP-based phylogeny of 35 ST38 *E. coli* genomes from the UK. The *bla*$_{OXA-23}$-positive isolates (n=3) were from our study, while the *bla*$_{OXA-23}$-negative isolates (n=32) were from Day et al. 2019[2]. The accompanying metadata alongside the phylogenetic tree contains information on the study, source, year, and sequence type (ST). The heatmap represents the genomic neighbourhood around *bla*$_{OXA-23}$ obtained through the short-read mapping approach, which includes the novel

composite transposon Tn*7816* and eight genes upstream and five downstream. In the heatmap, dark blue represents an identical sequence to the reference, while light blue indicates an absence of the sequence. Annotations at the top of the heatmap are represented by coloured arrows, with *bla*$_{OXA\text{-}23}$ represented in orange, ATPase in light green, IS*26* in light blue, other insertion sequences in dark blue, other genes in grey, and the eight genes located upstream and five genes downstream of Tn*7816* in white. The novel composite transposon Tn*7816* is visually indicated by red rectangle.

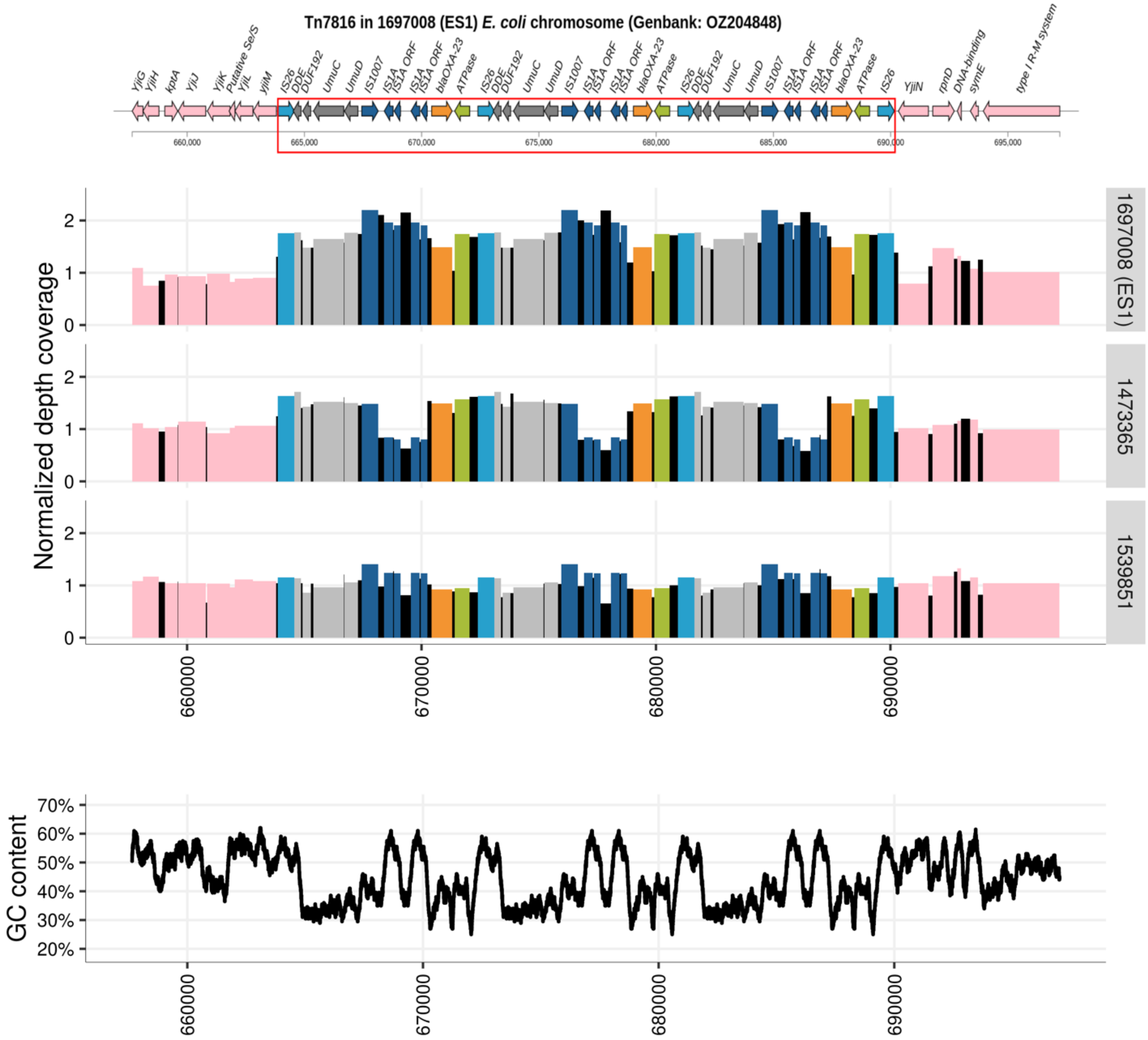

Supplementary Figure S3. Normalized depth coverage of short-reads and GC content of *bla*$_{OXA-23}$ genomic context in three UK *E. coli* isolates. This figure present three panels. The top panel schematically represents Tn*7816* carrying *bla*$_{OXA-23}$, plus upstream and downstream genes. Arrow colours correspond to specific genes. The middle panel shows the normalized short-reads depth coverage for each isolate, derived from mapping reads to the *E. coli* 1697008 (ES1) reference genome, with bar colours corresponding to genes and in black representing the intergenic regions. The bottom panel display the GC content distribution. For all panels, the x-axis represents nucleotide position on the *E. coli* 1697008 (ES1) chromosome.

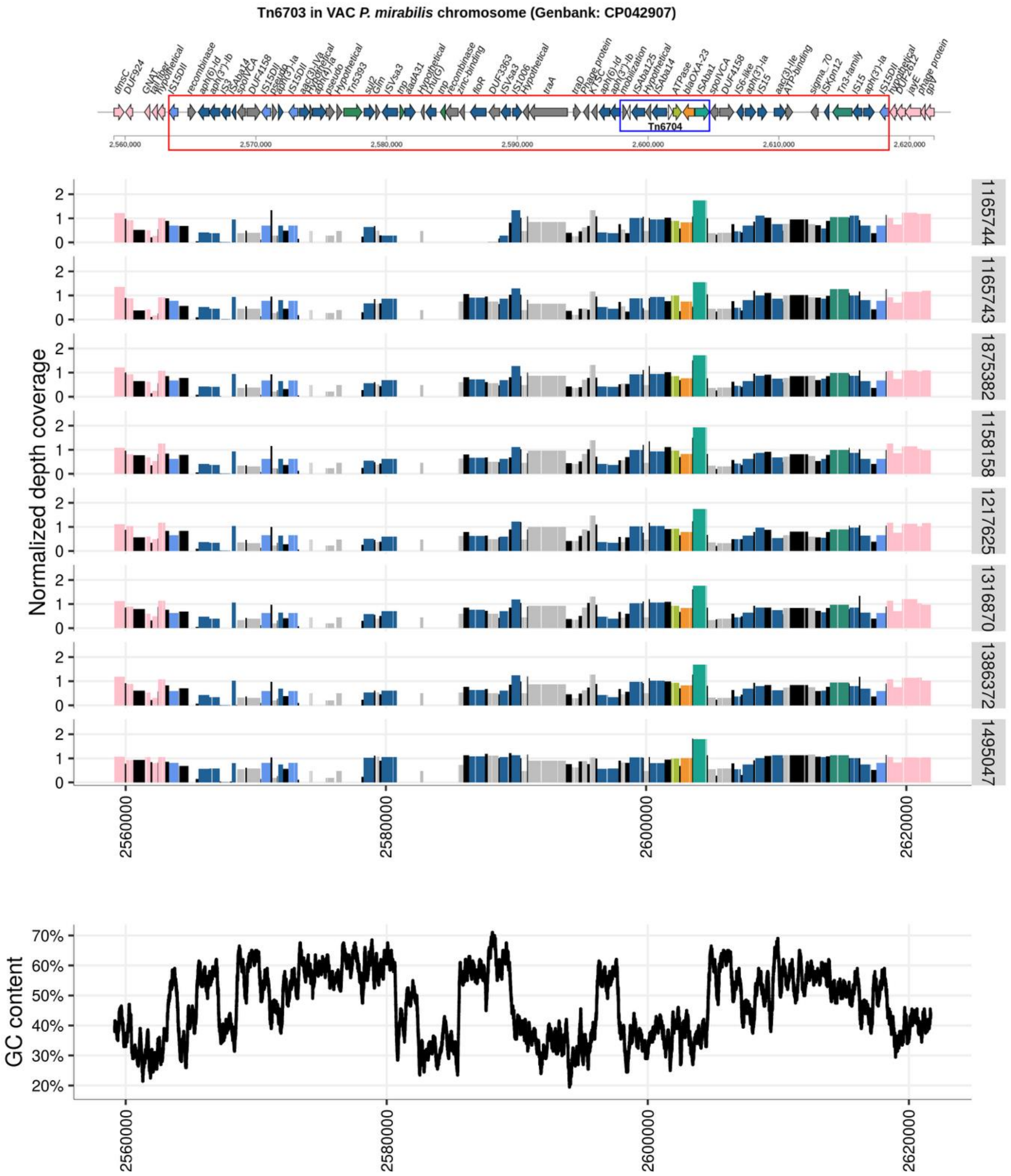

Supplementary Figure S4. Normalized depth coverage of short-reads and GC content of *bla*$_{\mathbf{OXA\text{-}23}}$ genomic context in eight UK *P. mirabilis* isolates. This figure present three panels. The top panel schematically represents Tn*6703* carrying *bla*$_{OXA\text{-}23}$, plus upstream and downstream genes. Arrow colours correspond to specific genes. The middle panel shows the normalized short-reads depth coverage for each isolate, derived from mapping reads to the *P. mirabilis* VAC reference genome, with bar colours corresponding to genes and in black representing the intergenic regions. The bottom panel display the GC content distribution. For all panels, the x-axis represents nucleotide position on the *P. mirabilis* VAC reference genome.

SUPPLEMENTARY TABLE

Supplementary Table S1. List of *P. mirabilis* (n=56) and *E. coli* (n=32) genomes from previous studies, including accession numbers and metadata. All *P. mirabilis* harbour *bla*$_{OXA-23}$, while none of the *E. coli* isolates do.

Isolate ID	Species	BioProject	BioSample	Assembly	SRA	Source	Country	Year	ST	Study
130B9	*P. mirabilis*	PRJNA521327	SAMN10883386	GCA_004570075.1		Human	France	2017	142	Bonnin et al. 2020[3]
160A10	*P. mirabilis*	PRJNA521327	SAMN10883387	GCA_004570785.1		Human	France	2018	185	
168F7	*P. mirabilis*	PRJNA521327	SAMN10883388	GCA_004570225.1		Human	France	2018	142	
172C2	*P. mirabilis*	PRJNA521327	SAMN10883389	GCA_004570215.1		Human	France	2018	142	
172J1	*P. mirabilis*	PRJNA521327	SAMN10883390	GCA_004570745.1		Human	France	2018	142	
175H8	*P. mirabilis*	PRJNA521327	SAMN10883391	GCA_004570715.1		Human	France	2018	142	
189B4	*P. mirabilis*	PRJNA521327	SAMN10883393	GCA_004570665.1		Human	France	2018	NA	
BCT11	*P. mirabilis*	PRJNA521327	SAMN10883407	GCA_004569245.1		Human	France	2017	NA	
BCT17	*P. mirabilis*	PRJNA521327	SAMN10883408	GCA_004569205.1		Human	France	2017	NA	
CNR20160617	*P. mirabilis*	PRJNA521327	SAMN10883403	GCA_004570145.1		Human	Belgium	2016	NA	
CNR20160679	*P. mirabilis*	PRJNA521327	SAMN10883401	GCA_004570175.1		Human	Belgium	2016	NA	
CNR20160877	*P. mirabilis*	PRJNA521327	SAMN10883402	GCA_004570235.1		Human	Belgium	2016	NA	
Cow-15-39117	*P. mirabilis*	PRJNA521327	SAMN10883396	GCA_004570065.1		Animal	France	2015	NA	
Dog-06-37660	*P. mirabilis*	PRJNA521327	SAMN10883397	GCA_004570055.1		Animal	France	2014	NA	
Dog-35-37761	*P. mirabilis*	PRJNA521327	SAMN10883398	GCA_004570195.1		Animal	France	2015	142	
GUI	*P. mirabilis*	PRJNA521327	SAMN10883404	GCA_004570115.1		Human	France	2016	NA	
L100	*P. mirabilis*	PRJNA521327	SAMN10883399	GCA_004570645.1		Human	France	2016	142	
L92	*P. mirabilis*	PRJNA521327	SAMN10883400	GCA_004570275.1		Human	France	2016	NA	
MOR	*P. mirabilis*	PRJNA521327	SAMN10883406	GCA_004569255.1		Human	France	2016	NA	
S4	*P. mirabilis*	PRJNA521327	SAMN10883394	GCA_004570775.1		Human	France	1996	NA	
VAC*	*P. mirabilis*	PRJNA521327	SAMN12566310	GCA_008041895.1		Human	France	2016	142	
Carb-01	*P. mirabilis*	PRJNA915754	SAMN32405799	GCA_030336315.1		Human	Germany	NA	142	Hamprecht et al. 2023[5]
Carb-13	*P. mirabilis*	PRJNA915754	SAMN32405811	GCA_030336105.1		Human	Germany	NA	142	
Carb-15	*P. mirabilis*	PRJNA915754	SAMN32405813	GCA_030336145.1		Human	Germany	NA	142	
Carb-17	*P. mirabilis*	PRJNA915754	SAMN32405815	GCA_030336065.1		Human	Germany	NA	135	
Carb-19	*P. mirabilis*	PRJNA915754	SAMN32405817	GCA_030336005.1		Human	Germany	NA	142	
Carb-21	*P. mirabilis*	PRJNA915754	SAMN32405819	GCA_030336025.1		Human	Germany	NA	142	
Carb-22	*P. mirabilis*	PRJNA915754	SAMN32405820	GCA_030335965.1		Human	Germany	NA	142	
Carb-23	*P. mirabilis*	PRJNA915754	SAMN32405821	GCA_030335945.1		Human	Germany	NA	142	
Carb-24	*P. mirabilis*	PRJNA915754	SAMN32405822	GCA_030335905.1		Human	Germany	NA	142	
Carb-28	*P. mirabilis*	PRJNA915754	SAMN32405826	GCA_030335865.1		Human	Germany	NA	142	
Carb-42	*P. mirabilis*	PRJNA915754	SAMN32405840	GCA_030335585.1		Human	Germany	NA	142	
Carb-43	*P. mirabilis*	PRJNA915754	SAMN32405841	GCA_030335605.1		Human	Germany	NA	142	
O87C3	*P. mirabilis*	PRJNA780406	SAMN23139460	GCA_021095805.1		Human	France	2019	NA	

O88A7	*P. mirabilis*	PRJNA780406	SAMN23139465	GCA_021095725.1		Human	France	2019	142	Lombes et al. 2022[4]
O88A8	*P. mirabilis*	PRJNA780406	SAMN23139466	GCA_021095705.1		Human	France	2019	142	
O88C6	*P. mirabilis*	PRJNA780406	SAMN23139468	GCA_021095635.1		Human	France	2019	142	
O88D1	*P. mirabilis*	PRJNA780406	SAMN23139469	GCA_021095615.1		Human	France	2019	142	
PmOXA23-1	*P. mirabilis*	PRJNA490489	SAMN10038483		SRS3767930	Human	France	2016	142	Potron et al. 2019[1]
PmOXA23-10	*P. mirabilis*	PRJNA490489	SAMN10038492		SRS3767937	Human	France	2017	142	
PmOXA23-11	*P. mirabilis*	PRJNA490489	SAMN10038493		SRS3767920	Human	France	2018	142	
PmOXA23-13	*P. mirabilis*	PRJNA490489	SAMN10038495		SRS3767923	Human	France	2018	142	
PmOXA23-14	*P. mirabilis*	PRJNA490489	SAMN10038496		SRS3767922	Human	France	2018	142	
PmOXA23-15	*P. mirabilis*	PRJNA490489	SAMN10038497		SRS3767926	Human	France	2018	142	
PmOXA23-16	*P. mirabilis*	PRJNA490489	SAMN10038498		SRS3767924	Human	France	2018	142	
PmOXA23-17	*P. mirabilis*	PRJNA490489	SAMN10038499		SRS3767925	Human	France	2017	142	
PmOXA23-18	*P. mirabilis*	PRJNA490489	SAMN10038500		SRS3767927	Human	France	2017	142	
PmOXA23-19	*P. mirabilis*	PRJNA490489	SAMN10038501		SRS3767919	Human	France	2017	142	
PmOXA23-2	*P. mirabilis*	PRJNA490489	SAMN10038484		SRS3767928	Human	France	2017	142	
PmOXA23-3	*P. mirabilis*	PRJNA490489	SAMN10038485		SRS3767931	Human	France	2017	142	
PmOXA23-4	*P. mirabilis*	PRJNA490489	SAMN10038486		SRS3767929	Human	France	2017	142	
PmOXA23-5	*P. mirabilis*	PRJNA490489	SAMN10038487		SRS3767933	Human	France	2017	142	
PmOXA23-6	*P. mirabilis*	PRJNA490489	SAMN10038488		SRS3767932	Human	France	2017	142	
PmOXA23-7	*P. mirabilis*	PRJNA490489	SAMN10038489		SRS3767935	Human	France	2017	142	
PmOXA23-8	*P. mirabilis*	PRJNA490489	SAMN10038490		SRS3767934	Human	France	2017	142	
PmOXA23-9	*P. mirabilis*	PRJNA490489	SAMN10038491		SRS3767936	Human	France	2017	142	
RH14000114	*E. coli*	PRJNA812750	SAMN26427511		SRS12339957	Human	UK	2013-2014	38	Day et al. 2019[2]
RH14000116	*E. coli*	PRJNA812750	SAMN26427512		SRS12339958	Human	UK	2013-2014	38	
RH14000211	*E. coli*	PRJNA812750	SAMN26426995		SRS12340023	Human	UK	2013-2014	38	
RH14000220	*E. coli*	PRJNA812750	SAMN26427015		SRS12340046	Human	UK	2013-2014	38	
RH14000221	*E. coli*	PRJNA812750	SAMN26426872		SRS12339696	Human	UK	2013-2014	38	
RH14000413	*E. coli*	PRJNA812750	SAMN26426928		SRS12340221	Human	UK	2013-2014	38	
RH14000447	*E. coli*	PRJNA812750	SAMN26427304		SRS12340175	Human	UK	2013-2014	38	
RH14000519	*E. coli*	PRJNA812750	SAMN26427770		SRS12340084	Human	UK	2013-2014	38	
RH14000531	*E. coli*	PRJNA812750	SAMN26427345		SRS12339839	Human	UK	2013-2014	38	
RH14000545	*E. coli*	PRJNA812750	SAMN26427625		SRS12339532	Human	UK	2013-2014	38	
RH14000550	*E. coli*	PRJNA812750	SAMN26426921		SRS12339535	Human	UK	2013-2014	38	
RH14000670	*E. coli*	PRJNA812750	SAMN26426945		SRS12339374	Human	UK	2013-2014	38	
RH14000703	*E. coli*	PRJNA812750	SAMN26426906		SRS12339495	Human	UK	2013-2014	38	
RH14000779	*E. coli*	PRJNA812750	SAMN26427420		SRS12339664	Human	UK	2013-2014	38	
RH14000823	*E. coli*	PRJNA812750	SAMN26427788		SRS12340104	Human	UK	2013-2014	38	

RH14000824	*E. coli*	PRJNA812750	SAMN26427783		SRS12340099	Human	UK	2013-2014	38
RH14000827	*E. coli*	PRJNA812750	SAMN26427784		SRS12340100	Human	UK	2013-2014	38
RH14000874	*E. coli*	PRJNA812750	SAMN26427399		SRS12339643	Human	UK	2013-2014	38
RH14001088	*E. coli*	PRJNA812750	SAMN26427580		SRS12340001	Human	UK	2013-2014	38
RH14001102	*E. coli*	PRJNA812750	SAMN26427025		SRS12340284	Human	UK	2013-2014	38
RH14001119	*E. coli*	PRJNA812750	SAMN26427734		SRS12339393	Human	UK	2013-2014	38
RH14001133	*E. coli*	PRJNA812750	SAMN26427303		SRS12340174	Human	UK	2013-2014	38
RH14001205	*E. coli*	PRJNA812750	SAMN26426978		SRS12339686	Human	UK	2013-2014	38
RH14001554	*E. coli*	PRJNA812750	SAMN26427341		SRS12339834	Human	UK	2013-2014	38
RH14001640	*E. coli*	PRJNA812750	SAMN26427586		SRS12340007	Human	UK	2013-2014	38
RH14001694	*E. coli*	PRJNA812750	SAMN26427359		SRS12339853	Human	UK	2013-2014	38
RH14001698	*E. coli*	PRJNA812750	SAMN26427055		SRS12339575	Human	UK	2013-2014	38
RH14002060	*E. coli*	PRJNA812750	SAMN26427331		SRS12339498	Sewage	UK	2013-2014	38
RH14002088	*E. coli*	PRJNA812750	SAMN26427561		SRS12340237	Sewage	UK	2013-2014	38
RH14002235	*E. coli*	PRJNA812750	SAMN26427380		SRS12339908	Human	UK	2013-2014	38
RH14002252	*E. coli*	PRJNA812750	SAMN26427050		SRS12339569	Sewage	UK	2013-2014	38
RH14002287	*E. coli*	PRJNA812750	SAMN26427711		SRS12339367	Sewage	UK	2013-2014	38

*The chromosome sequence for VAC isolate is available under the GenBank CP042907.1.

The “NA” label in the Year column indicates that the corresponding data was not available. Particularly, the German samples were collected between 2013 to 2022, however the specific collection year for each sample was not provided[5]. Similarly, the UK samples from Day et al. 2019[2] study were collected between 2013 and 2014, but information regarding the individual collection year for each sample was not available.

The "NA" in the ST column indicates that the sequence type could not be assigned due to the absence of necessary MLST loci, likely a result of fragmented genome assemblies.

SUPPLEMENTARY REFERENCES

1. **Potron A, Hocquet D, Triponney P, Plésiat P, Bertrand X, Valot B.** Carbapenem-susceptible OXA-23-producing *Proteus mirabilis* in the French community. *Antimicrob Agents Chemother* 2019; 63.
2. **Day MJ, Hopkins KL, Wareham DW, *et al.*** Extended-spectrum β-lactamase-producing *Escherichia coli* in human-derived and food chain-derived samples from England, Wales, and Scotland: an epidemiological surveillance and typing study. *Lancet Infect Dis* 2019; 19: 1325–35.
3. **Bonnin RA, Girlich D, Jousset AB, *et al.*** A single *Proteus mirabilis* lineage from human and animal sources: a hidden reservoir of OXA-23 or OXA-58 carbapenemases in Enterobacterales. *Sci Rep* 2020; 10: 9160.
4. **Lombes A, Bonnin RA, Laurent F, *et al.*** High prevalence of OXA-23 carbapenemase-producing *Proteus mirabilis* among amoxicillin-clavulanate-resistant isolates in France. *Antimicrob Agents Chemother* 2022; 66.
5. **Hamprecht A, Sattler J, Noster J, *et al.*** Proteus mirabilis – analysis of a concealed source of carbapenemases and development of a diagnostic algorithm for detection. *Clinical Microbiology and Infection* 2023; 29: 1198.e1-1198.e6.
6. **R Core Team. R:** A language and environment for statistical computing. 2021. Available at: https://www.r-project.org/.
7. **Cardona G, Mir A, Rosselló F, Rotger L, Sánchez D.** Cophenetic metrics for phylogenetic trees, after Sokal and Rohlf. *BMC Bioinformatics* 2013; 14: 3.
8. **Seemann T.** snippy: fast bacterial variant calling from NGS reads. 2015. Available at: https://github.com/tseemann/snippy.
9. **Schwengers O, Jelonek L, Dieckmann MA, Beyvers S, Blom J, Goesmann A.** Bakta: rapid and standardized annotation of bacterial genomes via alignment-free sequence identification. *Microb Genom* 2021; 7.
